# Effects of pre-pregnancy body mass index on pregnancy and perinatal outcomes in women with PCOS undergoing frozen embryo transfer

**DOI:** 10.1186/s12884-019-2611-1

**Published:** 2019-12-10

**Authors:** Jiaying Lin, Jialyu Huang, Ningling Wang, Yanping Kuang, Renfei Cai

**Affiliations:** Department of Assisted Reproduction, Shanghai Ninth People’s Hospital, Shanghai Jiaotong University School of Medicine, 639 Zhizaoju Road, Shanghai, 200011 People’s Republic of China

**Keywords:** Polycystic ovarian syndrome, Frozen embryo transfer, Obesity, Perinatal outcomes, Pregnancy outcomes

## Abstract

**Background:**

Previous studies have shown that among women with polycystic ovary syndrome who have difficulties conceiving, frozen-embryo transfer resulted in increased live birth rates and decreased ovarian hyperstimulation syndrome risk than did fresh-embryo transfer. In the present retrospective analysis, we sought to determine the effect of body mass index (BMI) on pregnancy and perinatal outcomes in women with PCOS undergoing FET.

**Methods:**

Women with PCOS (*n* = 1556) undergoing FET were divided into groups based on weight, with those with normal weight having a BMI of 18.5–24.9 kg/m^2^,those who were overweight having a BMI of 25–29.9 kg/m^2^, and those who were obese having a BMI ≥30 kg/m^2^. Both pregnancy and perinatal outcomes were compared among these groups.

**Results:**

The normal-weight, overweight, or obese groups exhibited similar pregnancy outcomes, including clinical pregnancy rate, miscarriage rate, ongoing pregnancy rate and live birth rate. In singletons, birth characteristics regarding newborn gender, gestational age, birthweight and length at birth were comparable between the three groups. For adverse neonatal outcomes, the three groups showed no significant differences on the rates of low birthweight, very low birthweight, preterm birth, and very preterm birth after adjustment. In addition, the obstetric complications and the frequencies of live-birth defects were also comparable between the three groups except that overweight and obese women were more likely than women of normal weight to have delivered via cesarean section.

**Conclusion:**

BMI did not affect the pregnancy or perinatal outcomes in women with PCOS undergoing FET.

## Background

Polycystic ovary syndrome [1] is an endocrine disease that is the most common driver of oligo/anovulatory infertility among women [2, 3]. Some studies suggest that PCOS may be more prevalent among overweight and obese women [4], but its incidence rate varies substantially across regions and ethnic groups [5]. The fertility and ovarian functionality of women with PCOS is influenced by a range of factors, with exhibiting higher levels of serum luteinizing hormone (LH), being overweight, and exhibiting hyperandrogenism (HA) all contributing to poorer fertility [6].

In-vitro fertilization (IVF) is increasingly being used for the treatment of infertility in women.

suffering from anovulatory forms of PCOS when induction of ovulation alone is insufficient to result in pregnancy, or when fertility is otherwise reduced [7]. However, there is limited data available regarding the effect of body weight on IVF outcomes in women who have PCOS.

With the refinement of vitrification techniques and ongoing adoption of single embryo transfer [8] strategies, the number of frozen embryo transfer [9] cycles has drastically increased. In countries that strongly favor SET policies, the proportion of FET treatments is as high as 50–80% [10]. One approach, known as a “freeze-all” strategy, involves cryopreserving all embryos, which are then transferred to a more physiological context during subsequent cycles. Previous studies have indicated that this freeze-all strategy results in a significant increase in the frequency of pregnancy as well as in live birth rates (LBR), in addition to significantly reducing the risk of ovarian hyperstimulation syndrome (OHSS) which can occur in patients undergoing IVF and ovarian stimulation, potentially leading to death [11, 12]. A randomized clinical trial (*n* = 1508) of women suffering from PCOS found that FET resulted in significantly increased live birth rates (49.3 vs 42.0%) and decreased OHSS incidence (1.3 vs 7.1%) relative to fresh embryo transfer [13]. To date, there has been only one published study investigating the correlation between elevated BMI and pregnancy outcomes in PCOS patients undergoing FET [14]. This previous study was limited by its small sample size, and its lack of follow-up regarding neonatal outcomes or birth defects. Therefore, in the present study we sought to assess the effect of maternal BMI on pregnancy and perinatal outcomes in women with PCOS undergoing FET.

## Methods

### Study patients

This study was conducted in the Department of Assisted Reproduction of the Ninth People’s Hospital of Shanghai JiaoTong University School of Medicine. Patients diagnosed as having PCOS according to the Rotterdam criteria who were aged ≥20 and < 35 years old who were undergoing their first FET cycles between June 2007and May 2017 were included in this retrospective cohort study. Neither assisted hatching nor preimplantation genetic screening (PGS) was performed in our center. Exclusion criteria included: 1) history of unilateral oophorectomy; 2) abnormalities of the uterus; 3) karyotypic abnormalities; 4) a history of multiple spontaneous miscarriages; 5) any conditions which precluded the safety of pregnancies or assistive reproductive technologies. Patient demographic information and cycle parameters were recorded in our medical records system. Our Hospital’s Ethics Committee approved this study.

### Measurement of BMI

BMI was calculated as follows: BMI = weight/height (kg/m^2^). We then used the standards determined by the World Health Organization to separate patients into three groups, with those with normal weight having a BMI of 18.5–24.9 kg/m^2^,those who were overweight having a BMI of 25–29.9 kg/m^2^, and those who were obsess having a BMI ≥30 kg/m^2^.

### Ovarian stimulation and laboratory protocols

All patients underwent one of the following three COH regimens: gonadotropin-releasing hormone antagonist (GnRH-ant), mild stimulation, or progestin-primed ovarian stimulation (PPOS). These regmins have been extensively described previously [15, 16]. Briefly, patients in the flexible GnRH-ant protocol were injected daily with 150–225 IU human menopausal gonadotropin (hMG; Anhui Fengyuan Pharmaceutical Co., China) from MC3, with daily 0.25 mg GnRH-ant (Cetrorelix, Mercerono) being initiated once the largest follicle was > 12–14 mm in size. For the mild stimulation protocol, patients received administration of 25 mg clomiphene citrate (CC; Fertilan, Codal-Synto Ltd., Cyprus) daily, with daily 2.5 mg letrozole (LE; Jiangsu Hengrui Medicine Co., China) started on MC3 and used for 4 days, whereas CC use was continued until trigger. Patients also received 150 IU hMG intramuscularly every other day starting on day MC6. For the PPOS regimen, patients were administered daily 10 mg medroxyprogesterone acetate (Shanghai Xinyi Pharmaceutical Co., China) and 150–225 IU hMG from MC3 to trigger day. Based on ovarian responses as assessed by transvaginal ultrasonography (TVU) and serum estradiol (E2) concentration, doses of hMG were adjusted. When a minimum of 3 follicles were at least18 mm in diameter, or a single follicle was at least20 mm in diameter, 1000–5000 IU human chorionic gonadotropin (hCG; Lizhu Pharmaceutical Trading Co., China) and 0.1–0.2 mg triptorelin were administered in order to trigger final oocyte maturation.

Once 34–36 h had passed following trigger administration, oocyte retrieval was conducted. A conventional IVF/ICSI approach was employed in order to fertilize the harvested oocytes in light of the specifics of a given semen sample. The zygotes were then transferred and cultured via Continuous Single Culture (Irvine Scientific, USA) for the entirety of the developmental stage. Three days after retrieval, embryos were graded as per Cummins’s criteria [17], and the best quality embryos (grade I -II) were selected for vitrification. Embryos that were of lower quality (grade III -IV) were subjected to extended culture, and morphologically good blastocysts (grade ≥ 3 BC) based on the Gardner and Schoolcraft scoring system [18] were selected for vitrification on day 5 or 6. The vitrification and thawing procedures were performed as in previous reports [15].

### Frozen embryo transfer

Endometrial preparation was conducted using a mild stimulation cycle or a hormone replacement therapy as described previously [19]. Follicular growth was, if needed, stimulated via administration of letrozole (5 mg) on cycle days 3–7, with follicular growth assessed from day 10 onwards. Where appropriate, patients were also administered hMG (75 IU/day) in an effort to further promote the growth of the follicle and the lining of the endometrium. Patients were administered 5000 IU hCG, and FET timing was determined as in previous reports [15]. Embryos or blastocysts were transferred into patients at a time point which resulted in appropriate endometrial synchronization. After patients were successfully determined to be pregnant, they were delivered a sustained progesterone dose until gestational week 10, and only those patients with endometrial membrane thinning during cycles underwent sustained hormone replacement. Beginning on day 3, patients were given ethinyl estradiol (75 mg/day) (EE; Shanghai Xinyi Pharmaceutical) orally in order to ensure that they exhibited endometrial thickening to a minimum of 8 mm, with evidence of a triple line pattern upon ultrasonic assessment. Once appropriate thickness was achieved, these patients received 0.4 g of progestin (Laboratoires Besins-Iscovesco) intravaginally each day, with embryo transfer being conducted after 3 days via abdominal ultrasonic guidance. In these patients, both estradiol and progestin dosing were maintained until gestational week 10.

### Pregnancy and perinatal outcomes

Rates of implantation were determined via ultrasound-mediated assessment of how many gestational sacs were evident in a patient as compared to the number of embryos transferred. Clinical pregnancy was established when there was a visible gestational sac present in the uterus after a 6–8 week period as determined via vaginal ultrasound. Rates of clinical pregnancy were determined by dividing numbers of clinical pregnancies by numbers of FET cycles. An ongoing pregnancy was one wherein a fetal heartbeat was detectable as of gestational week 12, with the number of such ongoing pregnancies being divided by the number of FET cycles to derive the ongoing pregnancy rate. Rates of miscarriage were determined based on the ratio of induced or spontaneous losses of pregnancy relative to clinical pregnancies. Live birth was defined as delivery of a living baby at ≥24 weeks gestational age. All other adverse outcomes, such as ectopic pregnancies, late miscarriages, stillbirths, or fetal defects were also monitored and recorded as appropriate. Obstetric outcomes included gestational hypertension, gestational diabetes, preeclampsia, premature rupture membrane and cesarean delivery. Outcomes among neonates included LBW (low birth weight: birth weight < 2500 g), very LBW (very low birth weight: birth weight < 1500 g), macrosomia (birth weight > 4000 g), PTD (preterm delivery:< 37 weeks of gestation), very PTD (very preterm delivery:< 32 weeks of gestation), small-for-gestational age (SGA: birthweight <10th percentile) and large-for-gestational age (LGA: birthweight >90th percentile).

When newborns died within 7 days following a live birth, this was recorded as an instance of early neonatal death for study purposes. In addition, any anomalous functional or genetic features of neonates or aborted fetuses were recorded. The International Classification of Diseases Q codes (Q00–Q99, 10^th^edition) were used in order to detect and classify any congenital deformities [20].

### Statistical analysis

For continuous variables, the normality was assessed via examining histograms and Q-Q plots as well as using the Shapiro-Wilk test. Data that were normally distributed were presented as means with standard deviations (SDs), while all other data were presented as medians (min - max). Continuous variables were compared via one-way ANOVAs, while categorical variables were compared via chi-squared tests. Multivariable logistic regression analyses were used to determine whether BMI was associated with rates of clinical pregnancy, ongoing pregnancy, miscarriage, live birth, PTD, very PTD, LBW and VLBW after adjusting for confounding factors, including maternal age, infertility duration, duration of cryopreservation, endometrial thickness, embryo quality, means of preparing the endometrium, number of embryos transferred, and embryo developmental stage. *P* < 0.05 was the threshold of significance. All statistical analyses were performed with SPSS for Windows v16.0(SPSS Inc., Chicago, IL, USA).

## Results

### Patient characteristics

Baseline characteristics of the patients in the three BMI groups are summarized in Table 1. We enrolled 1680 women in the present study, of whom 972, 480, and 228 were in the normal weight, overweight, and obese groups, respectively. Age, antral follicle numbers, infertility duration, previous IVF failures, types of infertility, types of FET cycles, endometrial thickness on transfer day, basal E2 levels, and indication proportions did not vary significantly among groups (*P* > 0.05) (Table 1). Consistent with the previous reports [21, 22], basal serum FSH, LH, and P levels were lower in overweight and obese women relative to those of normal weight (*P* < 0.001).

**Table 1:** Baseline characteristics of all FET cycles

Characteristic	Normal(N = 972)	Overweight(N = 480)	Obesity(N = 228)	Normal vs. Overweight	Overweight vs. Obestiy	Normal vs. Obesity
Age of embryo transfer (years), mean (SD),	32.82 ± 3.40	33.09 ± 3.81	33.27 ± 3.61	0.152	0.542	0.076
Duration of infertility (years), mean (SD)	3.80 ± 2.39	4.02 ± 2.80	4.21 ± 3.04	0.114	0.412	0.058
BMI of women, mean (SD)	21.45 ± 1.72	26.93 ± 2.21	32.13 ± 1.89	**< 0.0001**	**< 0.0001**	**< 0.0001**
Antral follicle count, mean (SD)	18.48 ± 6.23	18.85 ± 6.96	17.78 ± 8.01	0.306	0.069	0.218
Infertility causes, n (%)				0.075	0.968	0.727
PCOS only	266 (27.38%)	123 (25.72%)	59 (25.99%)			
PCOS + male factor	120 (12.32%)	55 (11.45%)	28 (12.19%)			
PCOS + tubal factor	486 (49.97%)	242 (50.34%)	115 (50.62%)			
PCOS + male factor + tubal factor	74 (7.69%)	33 (6.93%)	16 (7.21%)			
PCOS + other factor	26 (2.64%)	27 (5.56%)	10 (3.99%)			
Basal FSH (mIU/mL), median (min-max)	5.21 (0.32–9.98)	4.94 (0.26–9.99)	4.61 (0.45–9.99)	**< 0.0001**	**0.031**	**0.002**
Basal LH (mIU/mL),median (min-max)	5.36 (0.19–18.92)	4.57 (0.16–17.29)	4.02 (0.28–18.49)	**< 0.0001**	**0.031**	**< 0.0001**
Basal E2 (pg/mL),median (min-max)	36.00 (10.00–84.00)	37.00 (10.00–90.00)	37.00 (10.00–88.00)	0.546	0.929	0.618
Basal P (ng/mL),median (min-max)	0.30 (0.10–0.80)	0.30 (0.10–0.90)	0.30 (0.10–0.90)	**< 0.0001**	**0.032**	**< 0.0001**
Previous IVF failures n (%)				0.177	0.941	0.585
0	693 (71.28%)	361 (75.17%)	169 (73.96%)			
1–2	207 (21.28%)	94 (19.52%)	46 (19.99%)			
≥ 3	72 (7.44%)	25 (5.31%)	13 (6.05%)			
Type of infertility, n (%)	0.155	0.163	0.754			
Primary	652 (67.04%)	335 (69.72%)	156 (68.23%)			
Secondary	320 (32.96%)	145 (30.28%)	72 (31.77%)			
Type of FET cycle	0.112	0.073	0.062			
HRT	399 (41.07%)	217 (45.16%)	107 (46.93%)			
Ovarian stimulation	573 (58.93%)	263 (54.84%)	121 (53.07%)			
Endometrial thickness [17]	11.28 ± 2.02	11.32 ± 2.05	11.29 ± 1.98	0.532	0.329	0.835
Fertilization method,n (%)				0.523	0.822	0.894
IVF	562 (57.85%)	287 (59.71%)	131 (57.29%)			
ICSI	222 (22.82%)	97 (20.27%)	50 (21.96%)			
Half IVF + half ICSI	188 (19.33%)	96 (20.02%)	47 (20.75%)			

*PCOS* Polycystic ovary syndrome, *HRT* hormone replacement therapy, *ICSI* Intracytoplasmic sperm injection, *FSH* follicle-stimulating hormone, *LH* luteinizing hormone, *E2* estradiol; *P* progestogen. All *P* values were assessed using χ^2^ or ANOVA

*P* <0.05 was considered to be statistically significant

### Pregnancy outcomes

Our observed findings with respect to pregnancy outcomes in these three different BMI groups are described in Table 2. The normal, overweight, and obese groups of women exhibited similar rates of implantation (normal-weight: 41.08% vs. overweight:40.77% vs. obese: 38.52%,*P* > 0.05), clinical pregnancy (normal-weight: 59.26% vs. overweight:57.71% vs. obese: 55.26%,*P* > 0.05, miscarriage (normal-weight: 11.28% vs. overweight:16.61% vs. obese: 15.08%,*P* > 0.05, multiple pregnancies (normal-weight: 30.38% vs. overweight:33.21% vs. obese: 27.78%,*P* > 0.05), ectopic pregnancies (normal-weight: 1.56% vs. overweight:2.46% vs. obese:1.56%,P > 0.05), intrauterine and ectopic pregnancies (normal-weight: 0% vs. overweight:0.35% vs. obese:0%,P > 0.05), ongoing pregnancies (normal-weight: 54.94% vs. overweight:53.13% vs. obese: 52.19%,P > 0.05) and live births (normal-weight:50.21% vs. overweight:45.83% vs. obese: 46.49%,P > 0.05).

**Table 2:** Reproductive outcomes following transferring blastocyst stage embryo

Characteristics	Normal(*N* = 972)	Overweight(*N* = 480)	Obesity(*N* = 228)	Normal vs. Overweight	Overweight vs. Obestiy	Normal vs. Obesity
Number of FET	972	480	228			
Number of thawed embryos	1839	908	423			
Number of transferred embryos	1828	905	418			
Embryo quality,%(n)				0.329	0.593	0.443
High-quality embryos	1789 (97.26%)	877 (96.92%)	405 (96.81%)			
Low-quality embryos	50 (2.74%)	28 (3.08%)	13 (3.19%)			
Clinical pregnancy rate per transfer,%(n)	59.26%(576/972)	57.71%(277/480)	55.26%(126/228)	0.405	0.401	0.307
Implantation rate,%(n)	41.08%(751/1828)	40.77%(369/905)	38.52%(161/418)	0.476	0.325	0.282
Miscarriage rate,%(n)	11.28%(65/576)	16.61%(46/277)	15.08%(19/126)	0.069	0.432	0.184
Multiple pregnancy rate,%(n)	30.38%(175/576)	33.21%(92/277)	27.78%(35/126)	0.298	0.249	0.377
Ectopic pregnancy rate,%(n)	1.56%(9/576)	2.46%(7/284)	1.56%(2/128)	0.258	0.438	0.623
Intrauterine and ectopic pregnancy rate,%(n)	0%(0/585)	0.35%(1/284)	0%(0/128)	0.548	0.527	0.328
Ongoing pregnancy rate,%(n)	54.94%(534/972)	53.13%(255/480)	52.19%(119/228)	0.379	0.477	0.366
Live birth rate,%(n)	50.21%(488/972)	45.83%(220/480)	46.49%(106/228)	0.191	0.517	0.251

### Obstetrical and neonatal outcomes

Next, we examined obstetrical and neonatal outcomes among these three groups, revealing no significant differences with respect to adverse obstetric outcomes, gestational age at delivery, neonatal weight, neonatal length, or sex (Table 3). The only significant difference among groups was that overweight and obese women were more likely than women of normal weight to have delivered via cesarean section.

**Table 3:** Obstetrical and neonatal outcome stratified by BMI

	Singleton births			Normal vs. Overweight	Overweight vs. Obestiy	Normal vs. Obesity
	Normal(*N* = 341)	Overweight(*N* = 144)	Obesity(*N* = 79)			
Hypertensive disorders in pregnancy	7.62%(26/341)	9.72%(14/144)	10.13%(8/79)	0.295	0.551	0.317
Gestational diabetes	4.69%(16/341)	7.64%(11/144)	8.86%(7/79)	0.158	0.476	0.136
Preeclampsia	0.59%(2/341)	1.39%(2/144)	2.53%(2/79)	0.346	0.449	0.166
Premature rupture membrane	1.76%(6/341)	2.08%(3/144)	1.27%(1/79)	0.532	0.558	0.611
Birth weight, g						
VLBW (< 1500 g)	0.29%(1/341)	2.08%(3/144)	1.27%(1/79)	0.083	0.425	0.344
LBW (< 2500 g)	5.28%(18/341)	6.94%(10/144)	7.59%(6/79)	0.314	0.532	0.302
Macrosomia(> 4000 g)	4.39%(15/341)	9.03%(13/144)	8.86%(7/79)	0.052	0.589	0.113
Small-for-gestational age(<10th percentile)	6.15%(21/341)	7.64%(11/144)	7.59%(6/79)	0.352	0.607	0.409
Large-for-gestational age(>90th percentile)	18.47%(63/341)	19.44%(28/144)	18.99%(15/79)	0.463	0.547	0.519
Length at birth (cm)	50.13 ± 2.56	50.35 ± 2.73	49.78 ± 3.11			
Gestation weeks at delivery (weeks)						
Very PTD (< 32 weeks)	1.47%(5/341)	2.78%(4/144)	2.53%(2/79)	0.268	0.641	0.395
PTD (< 37 weeks)	7.62%(26/341)	11.11%(16/144)	10.13%(8/79)	0.168	0.516	0.317
Child’s sex, no.(%)						
Male	51.32%(175/341)	44.44%(64/144)	45.57%(36/79)	0.099	0.491	0.213
Female	48.68%(166/341)	55.56%(80/144)	54.43%(43/79)			

PTD (preterm delivery:< 37 weeks of gestation), very PTD (very preterm delivery:< 32 weeks of gestation), LBW (low birth weight: birth weight < 2500 g);VLBW (very low birth weight: birth weight < 1500 g)

### Congenital defects

As shown in Table 4, The most frequently encountered congenital defects in the maternal normal weight, overweight, and obese groups were circulatory system defects (1.10%),gastrointestinal tract congenital disorders (0.68%), and circulatory system defects (1.50%), respectively. Congenital defects were detected in 13/635 neonates (2.05%) in the normal weight group, in 2/296 neonates (0.68%) in the overweight group and in 2/133 neonates (1.50%) in the obese group. There were no significant differences in rates of these defects between groups, nor did these rates significantly differ among singletons, multiples, or as a function of neonatal sex among these three groups (*P* > 0.05). Types of malformations also did not differ significantly between the three groups (P > 0.05).

**Table 4:** Incidence of birth defects in live-born infants and type of malformations according to the classification from code Q00-Q99 in the international classification of Disease, tenth editon

Characteristics	Normal (All births *n* = 635)	Overweight (All births *n* = 296)	Obesity(*N* = 133)	Normal vs. Overweight	Overweight vs. Obestiy	Normal vs. Obesity
Number of birth defects, no. (%)	13 (2.05%)	2 (0.68%)	2 (1.50%)	0.101	0.369	0.507
Singletons, no (%)	10/341 (2.93%)	1/144 (0.69%)	1/79 (1.27%)	0.121	0.586	0.363
Multiples, no (%)	3/294 (1.02%)	1/152 (0.66%)	1/54 (1.85%)	0.581	0.460	0.495
Birth defects, by sex						
Male, no (%)	7/332 (2.11%)	1/141 (0.71%)	1/61 (1.64%)	0.263	0.517	0.642
Female, no (%)	6/303 (1.98%)	1/155 (0.65%)	1/72 (1.39%)	0.257	0.537	0.601
Detailed birth defects						
Q00-Q07 nervous system	0	0	0	/	/	/
Q10-Q18 eye, ear, face, and neck	1 (0.16%)	0	0	0.536	/	0.363
Q20-Q28 circulatory system	7 (1.10%)	0	2 (1.50%)	0.223	0.231	0.482
Q30-Q34 respiratory system	1 (0.16%)	0	0	0.536	/	0.363
Q35-Q37 cleft lip and cleft palate	0	0	0	/	/	/
Q38-Q45 digestive system	0	2 (0.68%)	0	0.241	0.718	/
Q50-Q56 genital organs	0	0	0	/	/	/
Q60-Q64 urinary system	3 (0.47%)	0	0	0.621	0.608	0.594
Q65-Q79 musculoskeletal system	1 (0.16%)	0	0	0.536	/	0.363
Q80-Q89 other malformations	0	0	0	/	/	/
Q90-Q99 chromosomal abnormalities	0	0	0	/	/	/

### Logistic regression assessment of pregnancy and neonatal outcomes as a function of BMI

Next, we performed a multivariate logistic regression analysis as a means of evaluating pregnancy or neonatal outcomes in the normal weight, overweight, and obese groups. As shown in Table 5, the odds ratio of the rates of clinical pregnancy, miscarriage, ongoing pregnancy, live birth, PTD, very PTD, and LBW were similar between the three groups after adjustment for covariates.

**Table 5:** Reproductive and neonatal outcomes for overweight and obese patients versus normal patients

	Overweight vs. normal weight	Obesity vs. normal weight
	Adjusted Odds Ratio (95% CI)	Adjusted Odds Ratio (95% CI)
Clinical pregnancy rate	0.93 (0.83–1.04)	0.47 (0.05–3.57)
Miscarriage rate	0.59 (0.15–2.35)	1.33 (0.88–1.98)
Ongoing pregnancy rate	0.96 (0.82–1.12)	0.96 (0.53–1.72)
Live-birth rate	1.05 (0.92–1.21)	0.62 (0.18–2.49)
PTD	1.36 (0.57–3.21)	0.93 (0.57–1.53)
Very PTD	1.13 (0.80–1.60)	0.81 (0.33–2.04)
LBW	1.15 (0.86–1.53)	0.67 (0.22–1.96)
Very LBW	0.92 (0.56–1.51)	1.08 (0.84–1.36)

PTD (preterm delivery:< 37 weeks of gestation), very PTD (very preterm delivery:< 32 weeks of gestation), LBW (low birth weight: birth weight < 2500 g);VLBW (very low birth weight: birth weight < 1500 g)

Analyses were adjusted for maternal age, infertility duration, duration of cryopreservation, endometrial thickness, embryo quality, means of preparing the endometrium, number of embryo transferred as well as embryo developmental stage

## Discussion

Obesity and PCOS are closely-related disorders with overlapping features [23], including possible negative effects on pregnancy or neonatal outcomes. In this large retrospective study of 1680 women with PCOS undergoing FET, we found for the first time women who were overweight or obese did not exhibit any significant differences in obstetric complications, pregnancy or neonatal outcomes such as very PTD, PTD, very LBW, LBW, macrosomia, SGA, LGA, or major congenital malformations relative to women of normal weight.

Previous studies that examined the impact of obesity on obstetric outcome have demonstrated an increased risk for of outcomes including hypertensive diagnoses, preeclampsia, gestational diabetes, operative delivery, and other adverse outcomes [24, 25]. In our study, we did not find any significant differences in maternal outcome between obese, overweight, and normal weight women undergoing FET, although normal weight women had a lower rate of cesarean section in comparison to the other groups. Nonetheless, the lack of statistical differences in our study could be due to the relatively small sizes in each group. The higher rate of caesarean sections may be partially related to the higher rate of complications in the obese/overweight women, however, there is data to suggest that this difference is not completely explained by the high-risk obesity-associated conditions alone [26]. High caesarean section rates have been documented in otherwise low-risk obese/overweight women, and it is therefore plausible that being obese/overweight itself is an independent risk factor for labor dystocia, resulting in increased caesarean section [27–29].

In recent years, some studies have examined the effect of BMI on pregnancy outcomes among women with PCOS following IVF, but the results remain controversial. For example, Akpinar found that in a study of 193 women with PCOS undergoing ovarian stimulation via the mid-luteal long GnRH agonist or flexible GnRH antagonist protocols in a fresh embryo transfer cycle, being obese or overweight had no effect on the implantation, clinical pregnancy, ongoing pregnancy, or miscarriage rates [30]. A separate Chinese study of 128 women undergoing a conventional long GnRH agonist protocol in a fresh embryo transfer cycle, however, found that being overweight (BMI ≥ 24 kg/m^2^) was linked to lower rates of live birth and miscarriage, although this trend was not significant, whereas the frequency of clinical pregnancies was significantly decreased in those with a high BMI relative to those with a normal BMI (26.5% vs. 46.8%, *P* = 0.022). A separate study of 398 women with PCOS combining FET and the GnRH-ant protocol determined that overweight/obese patients (BMI ≥ 24 kg/m^2^) exhibited significantly decreased rates of implantation, live birth, and birth of twins relative to normal weight controls, whereas rates of late abortion were significantly increased in these individuals with a higher BMI. There are many possible reasons for the variable results among studies, such as their limited samples sizes, or differences in methodology, study populations, adjustments for risk factors, or the use of fresh vs. frozen embryos, potentially leading to differences in outcomes. In one large retrospective study utilizing the Society for Assisted Reproductive Technology database to assess outcomes for a total of 45,163 cycles, a significant negative correlation between obesity and rates of pregnancy was identified only when autologous oocytes were used and not when donor oocytes were instead employed. These findings suggested that obesity tends to negatively affect embryo quality, while it produces no effect on the endometrial receptivity and early implantation, which is consistent with our findings that overweight and obese women with PCOS achieved similar pregnancy outcomes including the rates of implantation, clinical pregnancy, and live birth as did normal weight PCOS women undergoing FET, when the quality of thawed embryos transferred is similar in three groups. However, the miscarriage rate ranged from 11.28% in the normal BMI category to 16.61 and 15.08% in the overweight and obese groups (*P* = 0.069 and 0.184, respectively). It is likely that the increased miscarriage rates associated with an elevated BMI are related to insulin resistance. Altered plasma leptin levels and the leptin-resistant state associated with obesity has been shown to be associated with impaired trophoblastic invasion and early miscarriage [31–33]. In addition, plasma levels of leptin correlate with BMI and high leptin levels may exacerbate insulin resistance [8, 34], thus predisposing individuals to miscarriage.

Previous studies have thoroughly explored the effect of BMI on neonatal outcomes [35–37], with a recent meta-analysis of 46 studies determining that an elevated maternal BMI was linked to increased rates of fetal macrosomia, an elevated birth weight ≥ 90%of that of infants of equivalent gestational age, and a higher risk of premature birth for spontaneous conception. Nevertheless, there are only few studies focusing on the relationship between BMI and neonatal outcomes in the context of assisted reproductive technology [38, 39]. In 2016, Kawwass et al. reported that obesity was linked to an elevated risk of low birth weight and preterm delivery in the case of fresh autologous IVF cycles [38]. By retrospectively analyzing 354 singleton deliveries, a later study by Ben-Haroush et al. detected no variations in mean birth weight, gestational age, SGA, or preterm delivery as a function of maternal weight in women transferring a single fresh embryo, consistent with our study suggesting that there is no statistically significant impact of high BMI on the risk of delivering a preterm birth, or on low birthweight singletons in women with PCOS undergoing FET. Notably, previous studies were carried out in fresh IVF cycles and therefore, the possibility of harm induced by the supraphysiologic hormonal milieu or by suboptimal endometrial development during COH cannot be excluded. In contrast, FET can provide a physiological intrauterine environment, which is beneficial not only for endometrial receptivity and early implantation, but also for placentation and fetal growth, as evidenced by more favorable neonatal outcomes than fresh embryo transfer in previous studies [40]. In addition, our finding that pre-pregnancy BMI in women with PCOS was a predictor of macrosomia was consistent with previous studies [41–43]. Identification of women with PCOS undergoing FET at risk of macrosomia and targeted pre-conception interventions aimed at reducing body weight among women with a high BMI may mitigate these increases in macrosomia.

Among women with PCOS women undergoing FET, we found that the infants of overweight or obese women with PCOS had no elevated rates of congenital malformations as compared to those of women of normal weight. This is inconsistent with the results of previous studies of spontaneous conception [44, 45]. The Irish Atlantic Diabetes In Pregnancy (ATLANTIC DIP) study determined that among gluocse-tolerant women, obesity was still linked to an elevated incidence of birth defects [44]. Persson et al. reported that malformations of the genitals, nervous system, and digestive system were also increased in the offspring of obese mothers [45]. Watkins et al. observed higher rates of spinabifida, omphalocele, heart defects, and multiple anomalies when comparing neonates born to obese women relative to those born to women of a normal weight [46]. Unlike this past study, ours was among the first examining comparable outcomes in the context of a freeze-all-based FET approach in women with PCOS. As our data regarding congenital defects came from patient questionnaires, there is a risk that any minor defects may have been overlooked, although this is unlikely to have affected the overall study outcomes with respect to infant characteristics at birth.

A major weakness of this study is its retrospective format, and the potential heterogeneity in patient characteristics such as stimulation protocols used for ovarian stimulation or the presence of three different methods of endometrial preparation among patients. Although we adjusted our analyses to minimize the likelihood of confounding, it is impossible to completely preclude the possibility of underlying selection bias. In addition, our study was carried out over a long duration of more than 10 years in a single center. Thus, it will be interesting to validate these findings in a multi-center clinical trial over a shorter period of time in the future. Moreover, the data regarding the neonatal outcomes were derived from parental questionnaires and not from direct assessment of medical records, possibly leading to a failure to detect minor congenital defects, though any such defects were unlikely to markedly alter neonatal characteristics at birth.

## Conclusions

Our findings revealed that being obese or overweight is not significantly associated with increased risks of adverse pregnancy or neonatal outcomes. Further studies employing larger patient cohorts and extended follow-up durations will be necessary in order to verify the effects of high BMI on the pregnancy outcomes in women with PCOS, as well as to verify its long-term safety impact on neonates.

## Data Availability

The transcripts from which this manuscript was developed are available on request from the corresponding author.

## Abbreviations

AOR: Adjusted odds ratio
BMI: Body mass index
COH: Controlled ovarian hyperstimulation
FET: Frozen-embryo transfer
GnRH-ant: Gonadotropin-releasing hormone antagonist
HA: Hyperandrogenism
HMG: Human menopausal gonadotropin
IVF: In-vitro fertilization
LBR: Live birth rates
LBW: Low birth weight
LH: Luteinizing hormone
OHSS: Ovarian hyperstimulation syndrome
PCOS: Polycystic ovary syndrome
PGS: Preimplantation genetic screening
PPOS: Progestin-primed ovarian stimulation
PTD: Preterm delivery very
PTD: Very preterm delivery
SET: Single embryo transfer
VLBW: Very low birth weight
